# Size Matters: Genome Size Dynamics Driven by Copy Number Variation in a Green Alga

**DOI:** 10.1093/gbe/evad143

**Published:** 2023-08-08

**Authors:** Casey McGrath

*A new study challenges the conventional wisdom surrounding genome stability within closely related organisms and sheds new light on the mechanisms underlying extensive genome size variation.*

Our current understanding of genomic stability and variability has been largely informed by a few model organisms, making the extent and generality of these findings unclear. For example, although genomes vary greatly in size across the tree of life, exhibiting a more than 200,000-fold difference among eukaryotes alone, genome size is a feature that is generally considered to be stable within species or among closely related organisms. However, in a new study published in *Genome Biology and Evolution*, Takashi Tsuchimatsu and Yawako Kawaguchi from The University of Tokyo and their team uncover striking variation in genome size among the *Closterium peracerosum-strigosum-littorale* (*C. psl.*) complex, a group of unicellular algae closely related to land plants (Kawaguchi et al. 2023), challenging the traditional view of genomic stability.

**Fig. 1.**
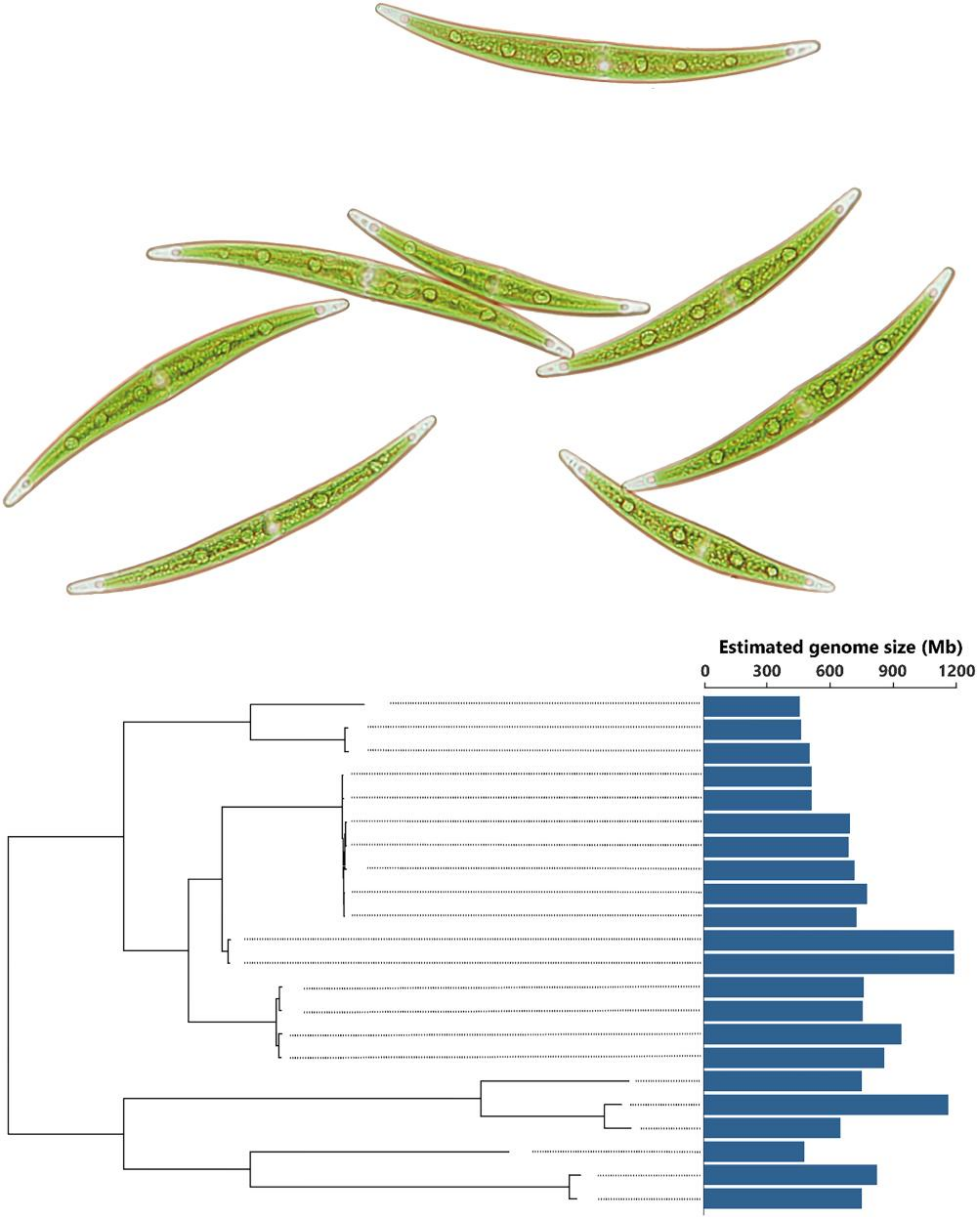
Members of the *C. psl.* complex, a unicellular Zygnematophycean alga, exhibit a more than 2-fold heritable variation in genome size. A photograph of *C. psl.* cells taken under the microscope is shown above a phylogeny of *C. psl.* strains and their associated genome sizes. Image courtesy of Yawako Kawaguchi.

According to Tsuchimatsu, the research team initially planned to conduct standard population and comparative genomic analyses of 22 natural strains of the *C. psl*. complex. However, “the genomes of *Closterium* appeared to be much more complex than we had initially thought,” says Tsuchimatsu. “Our most exciting finding was that there is extensive genome size variation between closely related algal strains that are morphologically indistinguishable.” Surprisingly, the *C. psl.* strains in the study exhibited a more than 2-fold variation in genome size, ranging from approximately 450 Mb to over 1,100 Mb (fig. 1). This discovery led the study's authors to shift the direction of the research in order to investigate the underlying factors contributing to such a considerable variation in genome size.

Generating genome sequence data from six additional *C. psl.* strains that differed markedly in the size of their genomes, Tsuchimatsu and colleagues further revealed that genome-wide copy number variation (CNV)—rather than duplication of specific chromosomes or proliferation of repeat sequences—plays a crucial role in driving the extensive genome size dynamics observed in this species complex. CNVs are the result of the duplication or deletion of genes or large DNA segments. The study's authors found that approximately 30% of genes varied in copy number, even among closely related *C. psl.* strains, suggesting that rapid changes in genome size were driven by frequent duplications and deletions that occurred across the genome.

Moreover, the researchers found that gene expression levels did not increase proportionally with gene copy number for about 30% of the genes exhibiting CNV. This suggests that an epigenetic (i.e., non-Mendelian process) like dosage compensation maintains balanced gene expression despite changes in gene dosage. As changes in gene dosage can be deleterious, dosage compensation may help preserve extensive CNV and genome size variation within the *C. psl.* complex. This sheds new light on the delicate interplay between gene copy number and expression levels, and it suggests that by increasing the tolerance for CNVs, dosage compensation may enable greater variation in genome size.

These revelations pave the way for additional research into genome dynamics in this species complex and among microeukaryotes in general. As noted by Tsuchimatsu, “Although we found signatures of extensive segmental duplications, we do not yet have a clear map of how duplicate sequences are distributed across chromosomes. For this, it will be necessary to obtain chromosome-scale assemblies together with basic karyotype information including chromosome numbers.” Unfortunately, “observing chromosomes at high resolution is still tricky in *Closterium*,” continues Tsuchimatsu, “and this presents a major obstacle.” Nevertheless, the research team has recently observed differences in chromosome numbers between *C. psl.* strains that can mate with each other, suggesting a mechanism tolerating chromosomal rearrangements during meiosis (Tsuchikane et al. 2023) and providing a tantalizing glimpse into additional genome dynamics in this species complex.

Further exploration in the *C. psl.* complex and other nonmodel species may continue to reveal the pervasiveness of such genome dynamics. For example, a study by Piganeau and colleagues that was also published recently in *Genome Biology and Evolution* revealed an unexpectedly high frequency of chromosomal duplications in experimental lines of unicellular green algae and found evidence for dosage compensation at the chromosomal level (Krasovec et al. 2023). Thus, by moving beyond observations in model organisms, these “exceptions” challenge the conventional rules of genomic stability and may ultimately reveal that eukaryotic genomes are much more dynamic than previously assumed.
